# The metabolic syndrome, thiazolidinediones, and implications for intersection of chronic and inflammatory disease

**DOI:** 10.1016/j.molmet.2021.101409

**Published:** 2021-12-01

**Authors:** Jerry R. Colca, Philipp E. Scherer

**Affiliations:** 1 Department of Biomedical Sciences, Western Michigan University School of Medicine, Kalamazoo, MI 49008, USA; 2 Cirius Therapeutics, Kalamazoo, MI 49007, USA; 3 Touchstone Diabetes Center, Department of Internal Medicine, USA; 4 Department of Cell Biology, University of Texas Southwestern Medical Center, Dallas, TX 75390-8549, USA

**Keywords:** Metabolic syndrome, NAFLD, COVID-19, TZDs, Insulin sensitizers, Mitochondrial pyruvate carrier

## Abstract

**Background:**

Chronic disease appears connected to obesity. However, evidence suggests that chronic metabolic diseases are more specifically related to adipose dysfunction rather than to body weight itself.

**Scope of review:**

Further study of the first generation “insulin sensitizer” pioglitazone and molecules based on its structure suggests that is possible to decouple body weight from the metabolic dysfunction that drives adverse outcomes. The growing understanding of the mechanism of action of these agents together with advances in the pathophysiology of chronic metabolic disease offers a new approach to treat chronic conditions, such as type 2 diabetes, fatty liver disease, and their common organ and vascular sequelae.

**Major conclusions:**

We hypothesize that treating adipocyte dysfunction with new insulin sensitizers might significantly impact the interface of infectious disease and chronic metabolic disease.

## 1 Background

The concept that metabolic dysfunction contributes to common diseases is not new. The observation that overnutrition has adverse effects on health goes back centuries. The anatomical observation of the simultaneous presentation of abdominal obesity and cardiovascular disease can be traced to the birth of anatomical pathology in 18th century Padua [1]. Modern studies have provided more color to the connection between overnutrition and diseases, variously described as under the umbrella of metabolic syndrome, which are all characterized by the reduced ability (resistance) of insulin to lower blood glucose [2]. There are several historical accounts of contributions that are defining the consensus views of the presentations of chronic metabolic disease that we know as fatty liver disease and diabetes (e.g., [3,4]). These concepts form the basis for the discussion that follows.

Although the concept of insulin resistance appears in older literature, the notion received greater attention in the 1980's when it became clear that cases of “adult-onset diabetes” or “type 2 diabetes” were characterized by reduced cellular response to insulin and increased circulating levels of insulin, rather than a primary loss of insulin as in “type 1” diabetes. These studies began to change the views of metabolic disease and the concepts of insulin resistance and insulin resistance syndrome gained general acceptance [5,6]. In that era, the Takeda company reported on empirically discovered compounds that lowered circulating glucose, lipids, and insulin [[7], [8], [9], [10]]. These compounds were categorized as thiazolidinediones (TZDs) based on the key structural feature of the TZD ring. In 1985, the Takeda-Upjohn partnership selected an analog called U-72107a (AD4833) or pioglitazone. These compounds became known as insulin sensitizers because they lowered plasma glucose in various diabetic animal models by reducing the resistance to insulin-stimulated glucose uptake [11]. As the initial compounds were discovered in phenotypic screens, the mechanism of action of these compounds was unknown. Uniquely, the pharmacology produced not only an improvement in glycemic control, but also a reduction in circulating insulin levels with preservation of pancreatic function. Given the promise of this pharmacology with the potential to impact the broad scope of metabolic disease, billions of dollars have been invested in an effort to find improved entities, however no improvements have made it to market. As reviewed previously [12,13], any attempt to develop new agents has been stymied by singular focus on the hypothesis that the nuclear transcription factor PPARγ was the obligate direct target of these compounds required for the pharmacology. The identification of a second target in mitochondria found by unbiased tracing of the binding of tritiated pioglitazone provides a new route to drug discovery [14]. As discussed below, the appreciation of this target also provides an overarching rationale for the potential of these agents to treat the overlapping pathology of chronic metabolic disease.

Pioglitazone (as Actos®), the third entry of the first generation TZDs to the market, became a successful treatment for type 2 diabetes although the dose and use of Actos® are both limited by its side effects. These limitations result from direct activation of transcription factors, particularly PPARγ, and this produced a significant roadblock to progress since, as discussed above, this transcription factor was thought to be the direct molecular target for the TZDs. In fact, rosiglitazone (Avandia®), the most potent marketed PPARγ agonist, has a less favorable safety profile than pioglitazone and, moreover, the intentional creation of directed potent activators came with even greater increase in fluid retention, edema, bone loss, and other issues that prevented further development. No improvements have progressed to the marketplace since Actos® was first approved in 1999 (reviewed in [12,13]).

Meanwhile, type 2 diabetes remains a growing issue in global health care. In the intervening years, there have been other types of agents approved for treating type 2 diabetes, including DPP4 inhibitors, SGLT2 inhibitors, and GLP1 receptor agonists, all of which have been introduced as second line agents after the treatment failure of diet, exercise, and metformin to adequately treat type 2 diabetes [15]. However long-term treatment of patients with type 2 diabetes often still requires the introduction of exogenous insulin to provide the best control of plasma glucose levels, once the initial treatment paradigms have failed. Moreover, in recent years, in spite of the availability of new agents, optimal control glycemia has not improved [16]. Some have argued for a broader use of pioglitazone because of its potential to prevent the loss of pancreatic β cell function and in particular, its efficacy in reducing cardiovascular disease, which remains the major cause of morbidity and mortality in both diabetic and prediabetic patients [17,18]. However, the use of pioglitazone remains limited by dose and tolerability to the treatment of type 2 diabetes as a second or third line therapy. The broader promise of these TZD agents has not been realized and there are no agents that have been approved to treat the other aspects of metabolic disease, the most common of which impact the physiology of the liver. This is particularly important, because the pathophysiology in the liver worsens, and likely in most cases precipitates, dysregulation of glycemic control eventually resulting in type 2 diabetes. Moreover, even in the absence of dysregulation of glycemic control, the fatty liver pathology exacerbates both hepatic and extrahepatic health care outcomes [[19], [20], [21]].

**Figure 1:**
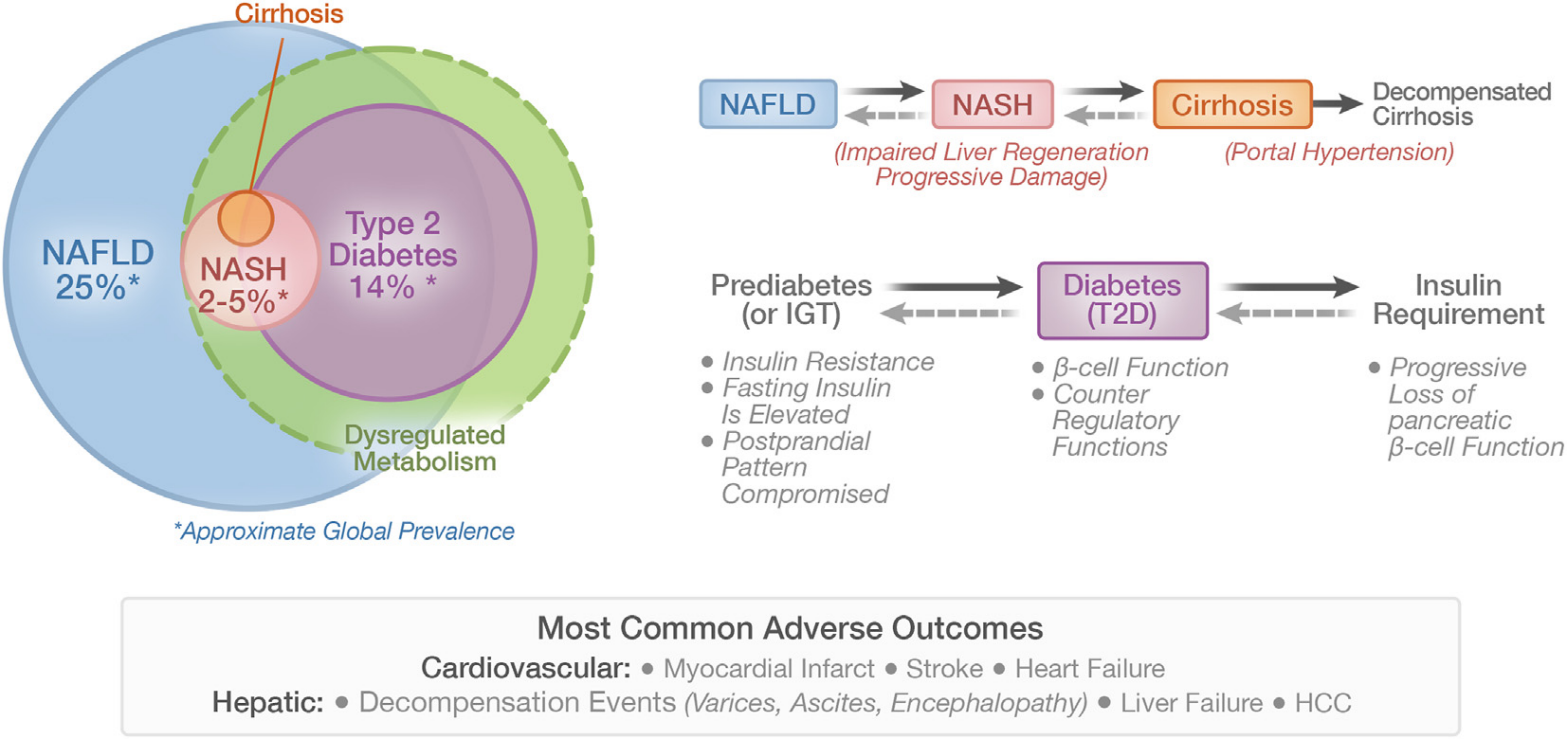
**Common chronic diseases of metabolic dysfunction.** Prediabetes-Diabetes and NAFLD-NASH-Cirrhosis are subsets of interconnected metabolic disease with significant global prevalence. These disease states are broadly impacted by environmental factors interacting with genetic predisposition. The primary precipitating factor is metabolic dysfunction/selective insulin resistance. Loss of glycemic control is precipitated by decompensation of pancreatic islet function resulting in the diagnosis of diabetes based on elevated plasma glucose. In a subset of individuals, progressive structural changes and loss of hepatic function results from ineffective repair/regenerative mechanisms. The definition NASH is based on pathology as read from liver biopsies. NAFLD/NASH and type 2 diabetes, whether diagnosed separately or as concomitant conditions, have common adverse cardiovascular outcomes. Liver-related events accelerate with increasing fibrosis (tissue damage) and loss of liver acinar structure (cirrhosis). In a subset of patients, portal pressure will continue to rise, resulting in clinical decompensation characterized by varices, hepatic encephalopathy, and liver failure.

There have been many reviews of non-alcoholic fatty liver disease (NAFLD), its interaction with type 2 diabetes, and the health care issues that it precipitates (e.g., [[22], [23], [24]]). The prevalence of NAFLD exceeds 25% of the global population and fatty liver disease can be progressive, leading to adverse outcomes involving the liver and other organ systems. NAFLD is generally recognized as a hepatic manifestation of metabolic syndrome and progresses at varying rates to cirrhosis, liver failure, or hepatocellular carcinoma, as well as extra-hepatic carcinomas. These issues, whether or not complicated by the co-diagnosis of type 2 diabetes, also predispose to a variety of cardiovascular sequelae [[19], [20], [21]]. Figure 1 shows the overlap of various degrees of metabolic liver disease and type 2 diabetes along with estimates of their global prevalence.

A potential timely route to approval of new drugs to treat aspects of the fatty liver syndrome, particularly as it relates to the hepatic sequelae, has been created by a histological definition of rapidly progressing NAFLD defined as non-alcoholic steatohepatitis (NASH). This subset of fatty liver disease is defined by histological measures that include biopsy-read endpoints of inflammation, specific types of cell death (described as ballooning) that can accompany the steatosis, and the severity has been further segmented by the level of fibrosis to define individuals who are at the greatest risk of progression to adverse liver outcomes. This definition has provided the basis of a potential subpart H route to regulatory approval based on the pathologists’ characterization. However, as proven in the process of multiple clinical trials with multiple agents, the usefulness of liver biopsies for drug development is limited by both sampling error (only a miniscule part of the liver is sampled) and the nature of the subjective assessment of a semiquantitative score [[25], [26], [27]]. This remains a significant barrier to drug development.

The pathology of NAFLD, and its more serious subset NASH, appears complex in that it involves many tissues and cell types and is characterized by insulin resistance, dysfunctional adipose tissue, and compromised immune function [28]. Interestingly, the overlapping characteristics of this pathology are all impacted by the so-called “insulin-sensitizing” pharmacology of pioglitazone [29,30]. Subjects with diabetes and hepatitis B, who are otherwise at high risk for decompensated cirrhosis and hepatocellular cancer, had significant reductions for both if they had been treated with pioglitazone [31]. However, as Alkouri and colleagues have reviewed, although pioglitazone might significantly impact the course of liver disease, the use of pioglitazone has been limited in these subjects, even in those with co-existing diabetes for which it is approved, because of issues with its tolerability [32]. A compound with this pharmacology that could be used in these subjects could have a substantial impact on health care outcomes from both hepatic and extra hepatic sequelae.

## 2 Selective insulin resistance

**Figure 2:**
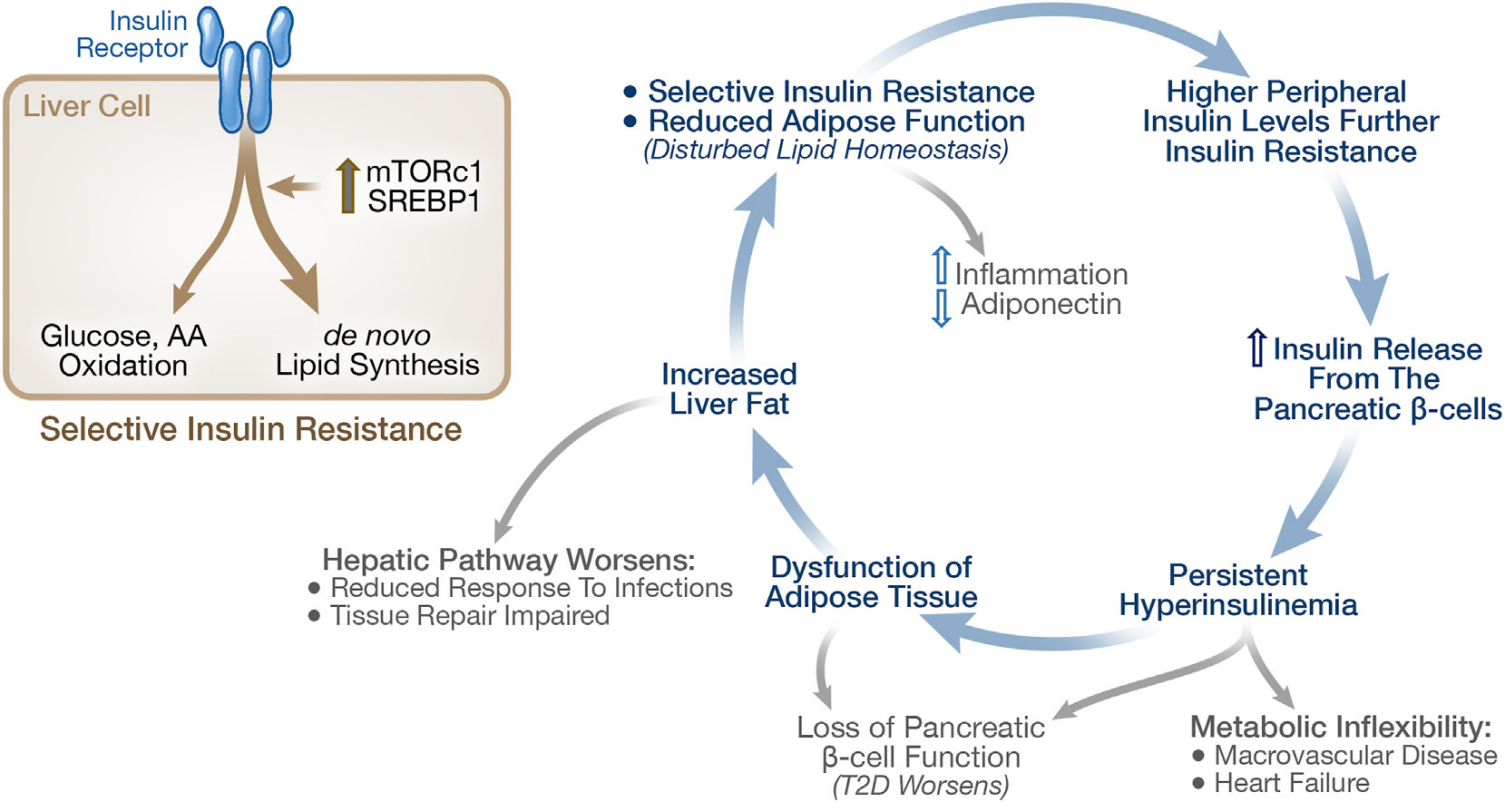
**Selective Insulin Resistance sets a vicious cycle of dysfunctional lipid metabolism.** Insulin resistance is selective, reducing the insulin action on glycemic control, while leaving the augmentation of *de novo* lipogenesis intact. This leads to a viscous cycle of peripheral hyperinsulinemia, greater insulin resistance, and eventual decompensation of the pancreatic beta cells in the subset of patients who progress to prediabetes and diabetes. The metabolic inflexibility that results from the loss of metabolic control directly contributes to the downstream pathologies with specific presentations modified by genetics and individual factors. The amount of ectopic lipid stored in the liver is related to disease severity. Reductions in ectopic fat in the liver associated with weight loss correlate with remission of type 2 diabetes.

Before proceeding with further discussion, it is important to consider the issue of “insulin resistance”, namely the reduced cellular response to the hormone insulin. As recently well-reviewed by Sbraccia and colleagues [4], it is important to understand that the insulin resistance associated with metabolic syndrome is not a general decrease in insulin action across the board - i.e., it does not affect all actions of insulin across all insulin sensitive tissues. Genetic studies provide insight into this issue. For example, genetic mutations in the insulin receptor that block insulin signaling produce very different syndromes than type 2 diabetes. Although these mutations result in reduced insulin action and in compensatory increased circulating insulin, these mutations do not result in hyperglycemia or diabetes. Furthermore, a series of elegant tissue-specific manipulations of the insulin receptor have shed more light on the role of the insulin receptor in various tissues, making it clear that most metabolic diseases are not the result of general generic insulin receptor defects. Importantly, the insulin resistance that occurs in the natural history of the metabolic syndrome is selective, reducing some aspects of insulin signaling, while leaving others to respond further to the increases in prevailing insulin levels. In the liver, insulin signaling pathways related to glycemic control and gluconeogenesis are impaired, while signaling pathways related to *de novo* lipogenesis and lipid storage, supported by activation of pathways, such as mTORC1 and SREBP1, are not [33,34]. As shown in Figure 2, this sets up a vicious cycle where selective insulin resistance results in more insulin release and higher insulin levels in turn drive more lipid deposition with a further decline in the function of adipose cells. This pathology can further the increase in peripheral insulin concentrations, since higher liver lipid also results in reduced hepatic clearance of insulin, resulting in higher peripheral insulin levels, blurring the change in insulin levels from fasting to postprandial conditions, and further reducing insulin sensitivity [35,36].

What causes the type of selective insulin resistance that defines the constellation of metabolic syndrome? The bulk of the data suggest that the root cause involves changes in lipid metabolism in multiple tissues. While there are diverse opinions on which lipid classes are most important in particular tissues, for example specific ceramides versus specific diacylglycerides (e.g., [37,38]), the driving force behind alterations of lipid metabolism in the key target tissues is a loss of functionality of adipose tissue, which in turn results in ectopic lipid accumulation in other cells and tissues. As reviewed in [4] and in support of this view, syndromes of lipodystrophy where the functionality of adipose tissue is compromised by genetic mutations, result in increased lipid content in peripheral tissues and extreme insulin resistance. On the other hand, overnutrition, although increasing storage of neutral lipid and expanding adipose mass, also results in metabolically inflamed, dysfunctional adipose tissue which further limits the capacity to maintain lipid homeostasis. Larger, lipid-laden adipocytes differ in function from smaller adipocytes for a variety of reasons, including hypoxia inducible gene regulation. These observations place the functional role of adipose tissue front and center in the generation of, and thus the consideration of, prevention of metabolic disease.

## 3 Adipose tissue and inflammation

In metabolic disease, increased inflammation in adipose tissue occurs with increased recruitment of macrophages, increased release of inflammatory cytokines, and reduction in adiponectin, an important anti-fibrotic mediator of homeostasis in peripheral tissues (reviewed in [[39], [40], [41]]). Dysfunctional adipose tissue results in disturbance of lipid homeostasis in peripheral tissues, resulting in selective insulin resistance. This has important implications in the insulin target tissues, such as the liver, but this also impacts pancreatic islets. Altered lipid metabolism together with loss of glycemic control results in loss of functionality of the insulin secretory cells and decompensation of the pancreatic control of glycemia (e.g., [42]). These observations support a unifying view that dysfunctional adipose tissue and changes in lipid metabolism contribute significantly to type 2 diabetes and metabolic syndrome through detrimental effects on multiple tissues.

The maintenance of a healthy adipose phenotype was one of the first events that was found in the development of pioglitazone, and it is this mode of action that likely contributes to the beneficial pleotropic actions of the drug. The previously overlooked mitochondrial molecular target for pioglitazone, the pyruvate carrier, provides a way forward to engage this mode of action to treat chronic metabolic disease without the side-effects that have limited the use of the first generation TZDs. As discussed below, rewiring pyruvate metabolism connects to cell fate and function through regulation of nutrient sensing mechanisms and the epigenetic regulation of gene transcription. Further definition of the downstream molecular actions should provide further rationale to explore the breadth of the TZD pharmacology.

## 4 New target for TZDs

**Figure 3:**
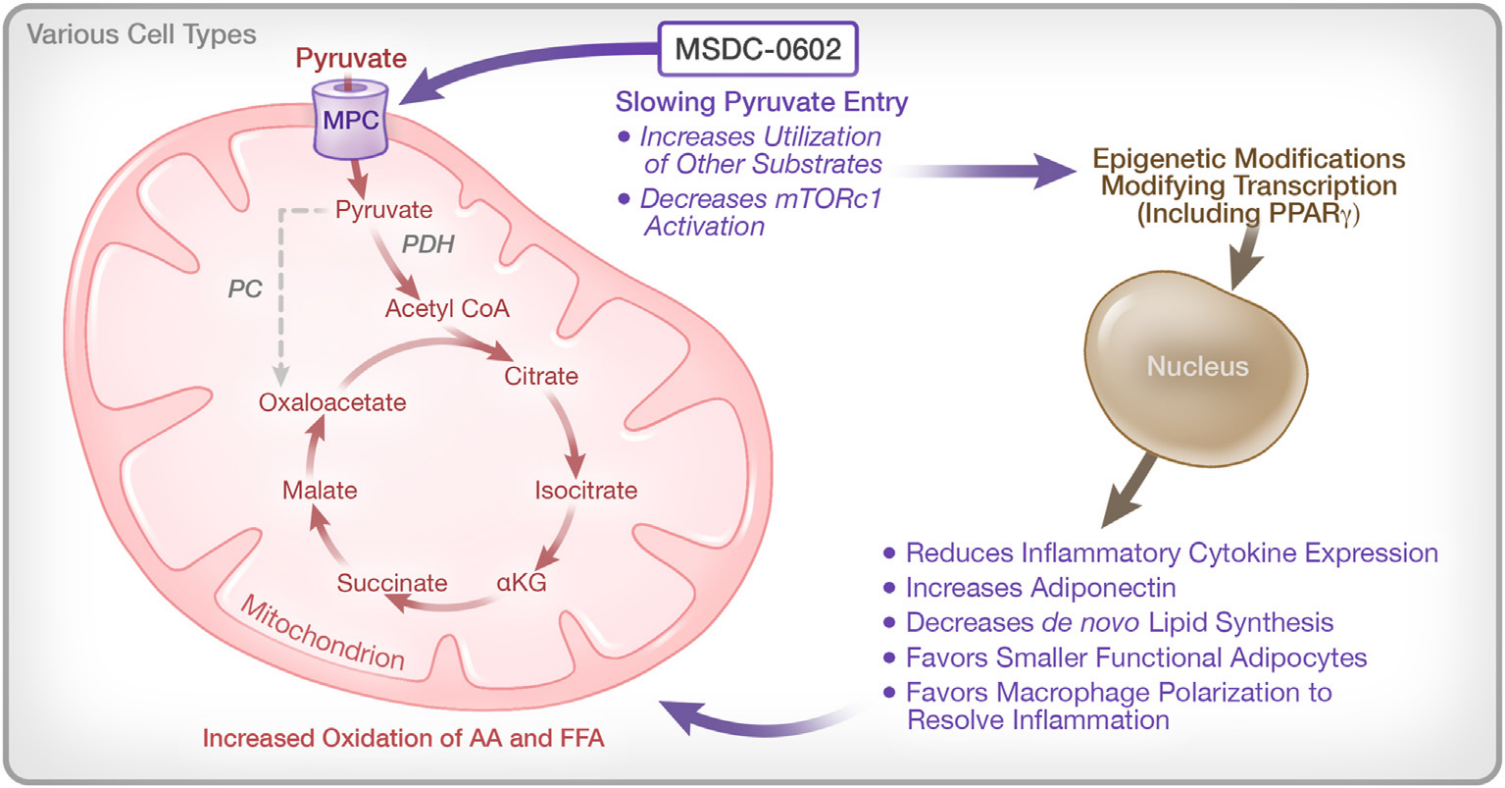
**Potential of the newly identified TZD mechanism to explain the pleotropic “insulin sensitizer” pharmacology.** Rewiring metabolism by slowing the entry of pyruvate into the mitochondrion alters the inflammatory response in multiple cell types with implications to chronic disease and response to environmental challenges such as viral infection. The general schematic of pathways that are involved is shown here. While many transcriptional networks are clearly involved, there has been a focus on PPARγ, since it is a master regulator of both adipose differentiation and the attenuation of the inflammatory response, both of which are known to be an important part of the TZD pharmacology.

As reviewed elsewhere, the early medicinal chemistry program in the Upjohn-Takeda collaboration to develop TZD analogs begin with empirical screens in animal models and culminated with the development of pioglitazone [12]. The work of Kletzien and colleagues [43,44] produced a cellular assay to predict the pharmacology of analogs that utilized the ability of these compounds to drive the differentiation of preadipocytes into a terminal adipose phenotype. Brown fat progenitor cells were the most sensitive to the effects of these analogs While this cellular phenotypic assay led to an early hypothesis that a direct effect on the transcription factor PPARγ, a master regulator of adipogenesis, might be the sole target for all of these analogs, it was subsequently found that there was not a complete agreement between the potency of the TZD analogs for binding to PPARγ and pharmacology [45,46]. The cellular assay based on phenotypic differentiation, rather than direct activation of PPARγ, was the best predictor of activity in the animal models of insulin resistance, which included KKAy mice, *ob/ob* mice, *db/db* mice, and mice fed a high fat diet. With this in mind, this functional differentiation assay was used to drive a search for new analogs, choosing only analogs that had been purposely *designed away* from direct binding to PPARγ principally by introducing polar substituents to reduced binding [47]. A TZD subset of molecules that had these properties still competed for the binding of tritiated pioglitazone to an unidentified target in the mitochondrial membranes. An original attempt to identify this target with ^125^I-photoaffinity drug analog crosslinking, misidentified the photoaffinity crosslinked protein as mitoNEET (CISD1). However subsequent studies properly identified the mitochondrial target of the TZDs as BRP44 (mpc2), an obligate component of the mitochondrial pyruvate carrier (MPC). The identification was proven by knockouts in both drosophila and a tissue specific knockout in mice [48,49]. All active TZDs, including pioglitazone and rosiglitazone, attenuate the uptake of pyruvate [50]. Liver specific knockouts of the MPC protect mice from diet-induced fatty liver [51]. The identification of the MPC as the mitochondrial target for the TZDs connects the pleotropic pharmacology of these molecules to alterations of mitochondrial pyruvate metabolism. Figure 3 summarizes some of the potential connections to the downstream pharmacology.

Genetic manipulation and inhibitor studies show that slowing entry of pyruvate into the mitochondria through the MPC results in an increase utilization of alanine and glutamine [52]. Thus, TZDs can impact the effects of overnutrition by rewiring metabolism. Studies conducted in *Drosophila* show that either TZDs or a knock down of the MPC ortholog counter the effects of a sucrose diet to increase or decrease the expression of a number of genes [49]. While all of the dots remain to be connected, the data suggest that the changes in metabolic programming conduct the pharmacology through modification of numerous downstream programs by undoing the effects of overnutrition, i.e., selectively changing the delivery of pyruvate to the oxidative cellular machinery. The downstream actions include reducing the degree of activation of mTORC1, an important regulator of autophagy, and a driver of the increase in *de novo* lipid synthesis in the presence of hyperinsulinemia. Ongoing studies are evaluating whether metabolic pathways also coordinate the epigenetic regulation of transcriptional networks, which can occur through reversable methylation of specific cytosines in DNA and through specific methylation and acetylation marks of key lysine within specific histone components of chromatin [53]. For example, it is known that certain histone marks can pause the differentiation of adipocytes [54] while modification through NAD^+^ generated by metabolism of α-ketoglutarate can remove these marks and allow differentiation to proceed [55]. The expression of adiponectin is limited by hypermethylation in obesity [56]. Recently *in vivo* and *in vitro* studies have shown methylation of DNA and histone modifications both connect changes in insulin signaling genes and PPARγ itself to insulin resistance [57,58].

## 5 New agents

MSDC-0602K is a new TZD developed on the rationale of selection of the MPC target over direct activation of PPARγ [59]. A recently completed Phase 2B dose-escalation study of MSDC-0602K in patients with biopsy-confirmed NASH with and without type 2 diabetes demonstrated full insulin-sensitizing pharmacology can be obtained with this agent without dose-limiting issues of pioglitazone [60]. The full effects of the one-year treatment of MSDC-0602K on lowering glucose, insulin, and liver enzymes in this study were seen at the middle dose (trough concentrations of approximately 4 μM of the active metabolite) and no dose-limiting effects were observed at a dose that produced twice these exposures over the one-year of the clinical trial.

While new agents being developed for the treatment of diabetes are evaluated and approved on the basis of glycemic control, development of agents for NASH is currently dependent on serial liver biopsies. As discussed above, given the technical issues involved with liver biopsies, including sampling error and subjective assessment of pathology, the development pathway for drugs to treat NASH has been complicated. It is possible that technical improvements could increase the utility of biopsies. However, biopsies will always be subject to sampling error [25,26]. Furthermore, any requirement for liver biopsies to gate and assess therapeutic effectiveness of marketed products would severely limit the use and utility of such agents. A successful solution to this conundrum will come from non-invasive/less invasive measures of the pathology (e.g., biomarkers and imaging) and ideally direct measures of liver function. In the meantime, avenues are being pursued which could facilitate the use of this new class of metabolic modulators such as MSDC-0602K in a broader application of metabolic syndrome to include liver disease. Brain penetrant analogs, such as MSDC-0160, are being evaluated for potential utility in the treatment of neurodegenerative disease [61]. The mechanism of action connects to the pathophysiology again through modifications of inflammation and autophagy [62,63].

## 6 Metabolic syndrome and host response to infectious disease

**Figure 4:**
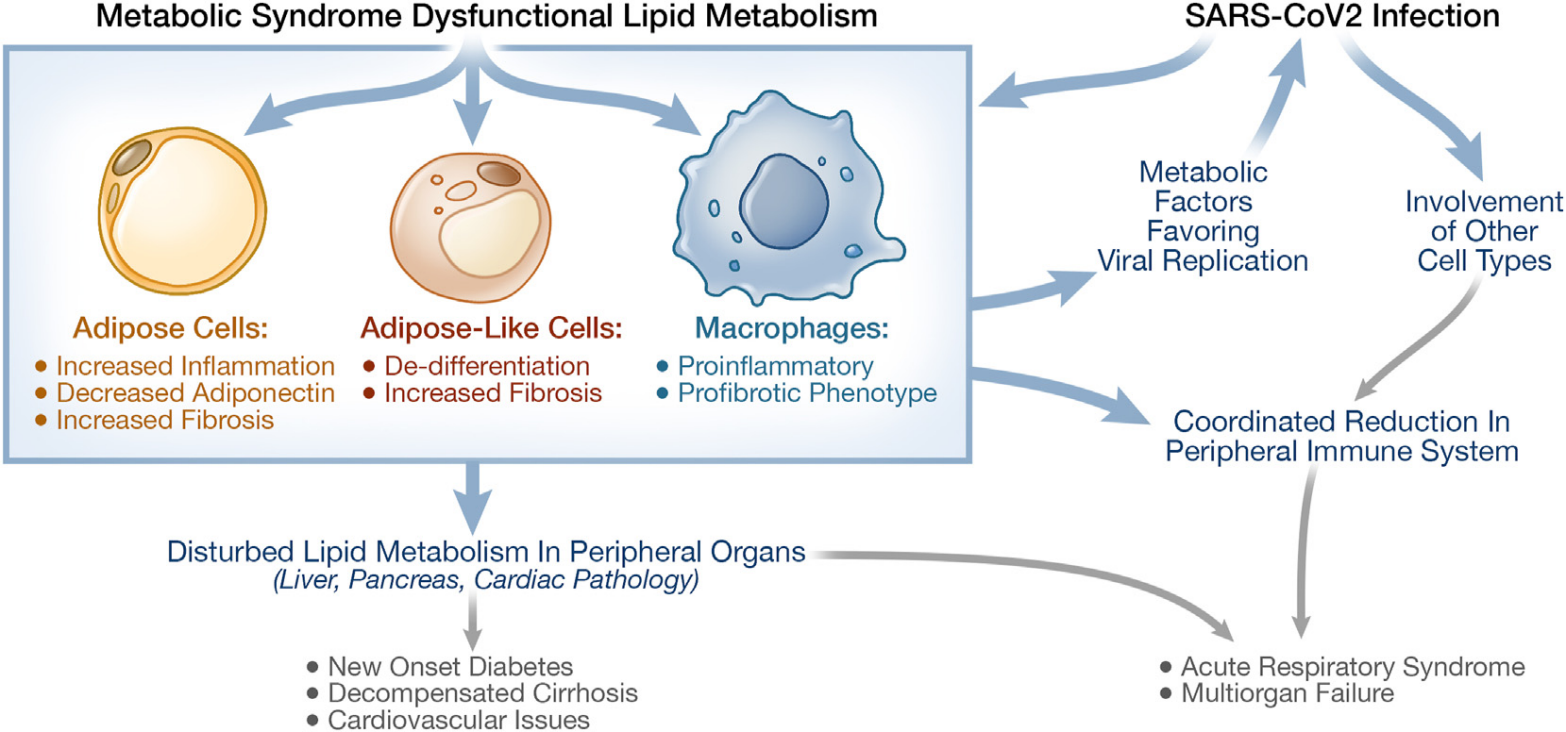
**Potential interactions of COVID-19 and metabolic disease.** Inflammation of adipose tissue that exist in metabolic disease worsens the viral infection and the response to the virus. The metabolic disturbance limits the recovery process and may facilitate colonization of other cell types as the virus propagates. The co-existence of the underlying metabolic dysfunction with the propagating infection can lead to increasing serious disease presentation. The longer term consequences are currently under intense scrutiny.

The winter of 2020 and the intervening months have led to a new understanding of the importance of treating metabolic disease in the face of an infectious disease. The adverse outcomes of COVID-19, the acute and post-acute consequences of the novel SARs-CoV2 coronavirus, appear to be driven primarily by host background [64]. While age of the subjects is clearly important, significance of age declines in subjects with a BMI>30, particularly if this increased body weight occurs together with hypertension, fatty liver, and diabetes [[65], [66], [67]]. Hyperglycemia is an adverse factor in subjects with or without diabetes [[68], [69], [70]]. There may also be a direct effect of viral infection of the pancreatic beta cells leading to the loss of beta cell function [71]. However, as with metabolic syndrome itself, dysfunction and inflammation in adipose tissue and the insulin resistance syndrome appear to be significantly associated with severity of disease. Acute infections result in a decrease in adiponectin expression and reduced circulating levels of adiponectin [72]. Data also show that severity of infection is accompanied by an increase in ectopic pancreatic and liver lipid as measured by MRI [73,74]. This is consistent with the hypothesis proposed by Scherer and colleagues that increased fibrosis and inflammation secondary to reduced adipose-like phenotype in multiple tissues, including the lung, are probably important in the host response [75]. Some potential interactions between metabolic disease and SARS-CoV-2 infection are shown in Figure 4. Adipose inflammation and reduced adiponectin levels have also been implicated in response to H1N1 viral infections [76]. While these data suggest that inflammatory adipose tissue is a major culprit, differential responses of the immune system is also likely to be a key component.

## 7 Metabolism, immune function, and tissue repair

Host immune cells gate the response to infectious agents with macrophages playing a central role. *Batabyal* et al. have reviewed the connection between metabolic dysfunction and immunometabolism as it relates to COVID-19 pathology [77]. Key findings have direct application to the TZD pharmacology. Elevated tissue and circulating levels of cytokines, particularly MCP-1 (CCL2), and reduced numbers of circulating leukocytes relative to neutrophils, correlate with the severity of COVID-19 pathology [78]. This suggests poorer outcomes may result from an imbalance between the innate and adaptive immune responses. Transcriptional analysis of macrophages from the lungs of patients with severe COVID-19 present with a proinflammatory and profibrotic signature [79]. *Desterke* et al. have shown that this proinflammatory and profibrotic phenotype of macrophages results in part from the downregulation of PPARγ through epigenetic mechanisms [80]. These data fit with the work of *Sun* et al. who have shown the importance of PPARγ networks in macrophages in both the initial responses and recovery to viral infections and to longer term repair mechanisms following the resolution of an infection [[81], [82], [83]]. The same pathways are upregulated by the metabolic actions of the TZDs and may also have broader implications to tissue regeneration and repair (see Figure 3, Figure 4).

*Fadini and colleagues* have studied how the release of hematopoietic stem/progenitor cells (HSPCs) from the bone marrow is impaired in subjects with diabetes and have postulated that this may be involved in impaired homeostasis [84]. They serendipitously observed that release of these progenitor cells into the circulation in response to an acute stimulus was uniquely returned toward normal in diabetic patients who were being treated with pioglitazone. This phenomenon carried over to animal models and could be traced to an activation of downstream PPARγ-regulated pathways in bone-derived macrophages which then coordinate the response of other cells. Consistent with the dynamic role of metabolism on inflammation, elevation of fructose in culture medium can itself directly reprogram macrophages to a proinflammatory state *in vitro* [85]. Thus, metabolically regulated pathways can impact immune function and tissue repair and may contribute to the host response, putting individuals who have chronic metabolic disease at a higher risk of adverse outcomes from infectious disease.

## 8 Weight lost and pharmacological treatment

Weight loss is the most direct approach to impacting metabolic disease. *Lean, Taylor, and colleagues* [86,87] have demonstrated effective weight loss treatment can actually result in the reversal of type 2 diabetes and allow the discontinuation of therapies for glycemia and hypertension. In the primary care-based Diabetes Remission Clinical Trial (DiRECT) trial, substantial weight loss led to remission of type 2 diabetes in a significant number of subjects (46% at 12 months and 36% at 24 months). When remission occurred, it correlated with a substantial reduction in ectopic lipid in the liver but required the return of normal pancreatic β−cell function. These data are consistent with the hypothesis that dysfunctional homeostasis of lipids can precipitate multiple pathologies [88]. Weight loss is not a complete solution to the health care dilemma however, since many subjects cannot maintain the life-style change and a subset of patients remain unresponsive. Bariatric surgery is another approach to impact metabolic disease through limiting caloric intake and this also has efficacy in reducing diabetes and fatty liver. Interestingly, a retrospective examination of metabolic surgery patients with COVID-19 in the Cleveland Clinic system who demonstrated weight loss and metabolic improvement had lower rates of hospital and ICU admission [89]. It is also possible to achieve weight loss with various peptide treatments, such as incretin GLP1-R agonists, which have been approved for use for treatment of diabetes and more recently for weight loss (reviewed in [90]). Other combination peptide agonists, e.g., GLP1 and GIP and GLP1 and glucagon (similar to the gut hormone oxyntomodulin) are in development. These treatments tend to be expensive and have initial gastrointestinal side effects. Moreover, while all of these approaches to weight loss can impact metabolic disease at a broad level, these approaches do not address the acute issues at the intersection of chronic metabolic disease with infectious agents, such as SARS-CoV2. On the other hand, the liabilities of metabolic disease on health care outcomes can be acutely impacted by addressing metabolic liabilities in both the adipose tissue and immune regulation by reprogramming metabolism even in the absence of weight loss.

## 9 Place for the new TZD pharmacology

Mitochondrially acting TZDs can impact the spectrum of metabolic disease without the dose-limiting effects of the first generation TZD insulin sensitizers. The tendency to produce weight gain, albeit reduced from high dose pioglitazone, is a TZD action that cannot be eliminated by selection against direct activation of PPARγ. On the other hand, as reviewed within, the learning of this pharmacology over the last three decades suggests that the TZD pharmacology uncouples the adverse effects of overnutrition from the dysfunction that would otherwise occur involving insulin target tissues, the pancreatic islets, and the immune system and that these effects can favorably impact interrelated chronic diseases of metabolism. Consistent with these observations, a *post hoc* analysis of the potential of pioglitazone treatment to prevent cardiovascular disease demonstrated that the greatest effect occurred in those subjects who gained weight over the course of the PROActive trial [91]. Moreover, genetic studies indicate that overweight subjects who are not insulin resistant actually have reduced incidence of cardiovascular disease [92]. Experience with MSDC-0602K suggests that whether subjects gained or lost weight did not impact the degree of benefit in terms of glycemic control or reduction of liver enzymes [60]. Thus, although this pharmacology uncouples body weight from these metabolic disorders, weight loss could still be pursued by adjusting diet, altering the dosing schedule, or combination with other agents which could provide additional benefit. For example, preclinical studies show very interesting effects with this agent in combination with the GLP1 agonist liraglutide [93] and with omega-3 fatty acids [94], paving the way for explorations of combination treatment in the clinic. Importantly, as evidenced by the pandemic in 2020–2021, acute interventions may be important and the TZD pharmacology is unique in its potential to intervene acutely at the intersection of dysfunctional adipose tissue and immune response in challenges, such as COVID-19. Recently, a multinational retrospective examination of records of COVID-19 cases across 56 heath care groups demonstrated that prior use of GLP-1 agonists and/or pioglitazone was associated with reduced hospital admissions [95]. While the full effectiveness of GLP1 receptor agonists may require time to impact the loss of weight, a TZD might be able to provide more immediate prospective benefit.

## 10 Conclusions

In conclusion, further study of the TZD clinical pharmacology will help restore the promise of a more effective treatment for chronic disease. Connecting the dots as to how modulation of pyruvate metabolism by TZDs results in the pleotropic pharmacology in multiple cell types should lead to an improved understanding of common chronic metabolic disease and more optimal use of such agents. We hypothesize that this pharmacology might also prove useful at the intersection of chronic metabolic and infectious disease for which there is a dire need during the current pandemic.
